# Artificial Intelligence Chatbots as Virtual Patients in Dental Education: A Constructivist Approach to Classroom Implementation

**DOI:** 10.1111/eje.13135

**Published:** 2025-06-03

**Authors:** Bree Jones, Aditya Desu, Christopher D. F. Honig

**Affiliations:** ^1^ Melbourne Dental School University of Melbourne Carlton Victoria Australia; ^2^ Business Services University of Melbourne Carlton Victoria Australia; ^3^ Department of Chemical Engineering, Faculty of Engineering and IT University of Melbourne Carlton Victoria Australia

**Keywords:** chatbot, constructivism, dental education, generative artificial intelligence, simulation

## Abstract

**Introduction:**

Advances in artificial intelligence (AI) have led to new possibilities for AI role‐play in classroom settings, where generative pre‐trained transformers (GPTs) and chatbots can potentially simulate interactions with patients. This pilot study aimed to design an AI role‐play activity underpinned by constructivist principles, implement the activity in a classroom setting, and evaluate the students' engagement with the activity and usability of the interface.

**Materials and Methods:**

The AI role‐play was designed based on a patient presenting to a dental teaching hospital in pain. It comprised two interconnected chatbots intended to simulate a patient consultation and clinical supervisor discussion. The chatbots were built using the open‐source framework Streamlit and powered by Chat GPT‐4. Second and final‐year students from an oral health degree were recruited through convenience sampling. Classroom observations were recorded and final‐year students participated in a usability questionnaire to gain insights into their engagement, technical challenges, and suggestions for improvement. Data usage and token costs were collected to assess the AI chatbot's feasibility.

**Results:**

Educator observations noted that AI role‐play facilitated peer discussion, highlighted gaps in history‐taking, and promoted peer learning. Usability survey feedback (*n* = 14) suggested that students perceived the AI role‐play as authentic and relevant to clinical practice, with 86% strongly agreeing it aligned with real‐world dental scenarios. The screen design, navigation, and multimedia integration were highly rated. The approximate costs for the activities were $13.17 (USD) for approximately 20 queries, per 20 students.

**Conclusion:**

This study presents a descriptive analysis of the implementation of an AI role‐play within the early phases of a design‐based research framework. AI role‐play activities can be implemented in a classroom setting and are perceived as useful for students to apply skills in taking a pain history. While results showed promise for scalability and authentic learning, future research evaluating the impact of the implementation of these activities on student learning should be explored.

## 1 Introduction

Generative pre‐trained transformers (GPTs) and chatbots have been used in tertiary education even before the public release of ChatGPT‐3 [1]. However, since its release, there has been a notable increase in interest in the broader application of generative artificial intelligence (GenAI) in tertiary education [2, 3, 4, 5, 6]. GenAI refers to AI systems capable of creating new content, including text, images, and simulations. This surge in interest is driven by the potential advantages these technologies offer, including enhanced accessibility to education, scalability for online learning environments, immediate responses, personalised learning experiences, and reduced operational costs [7].

The rapid growth of literature on GenAI in tertiary education has made it challenging to stay fully aware of all new developments. Much of the literature has explored opportunities and risks associated with GenAI, particularly concerning academic integrity within tertiary education [2, 3, 5, 8, 9]. More recently, discussions on GenAI in tertiary education have expanded to encompass broader applications, now spanning educational and administrative functions within universities. For instance, GPTs like ChatGPT have been leveraged to create innovative teaching methodologies [10, 11] and simplify operational tasks such as designing course learning objectives [12]. Furthermore, GPT‐powered tools are increasingly being explored to automate student assessment and feedback processes [13, 14, 15].

Beyond their operational roles in tertiary education, GPT‐powered chatbots show significant potential in providing direct instructional support to learners [16]. A promising application is AI tutors, such as Khan Academy's Khanmigo platform. These AI tutors can engage students in discussions on complex curriculum content using plain language and adapt their teaching styles to cater to individual learning needs. Their typical functions include precise information retrieval from course materials, study coaching, and interactive knowledge transfer [16, 17].

However, a significant challenge for AI tutors involves “hallucinations” from large language models (LLMs): incorrect or nonsensical responses that deviate from factual accuracy. This issue often stems from limitations in the model's training data, misinterpretation of user input, or incomplete and ambiguous inputs [18].

Two primary learner expectations have emerged when designing AI tutors, akin to expectations for certified course materials or professional teaching staff [19].

1. *Reliability (Performance Expectancy)*: Learners expect AI tutors to provide information reliably, similar to verified and accredited teaching materials. However, current GPT models may not consistently meet this standard. This contributes to significant learner distrust in their reliability for educational purposes and is a barrier to greater uptake [19].
2. Compellability: Learners expect AI tutors to consistently provide answers to course‐related questions, even when these questions lack full context. This demand requires AI tutors to deliver accurate responses consistently, regardless of ambiguous or extraneous queries [19]. This expectation surpasses the allowances typically granted to human educators, who can admit they do not know, state that a question is outside of the course scope or may understandably make occasional errors.

These expectations raise important questions about the reliance on AI tutors for fact retrieval and the potential limitations of such an approach.

An emerging and compelling use case for GenAI chatbots is AI role‐play. Unlike AI tutors, AI role‐play involves chatbots simulating personas or scenarios to provide authentic workplace experiences. For instance, GPT chatbots have been used to simulate student interactions for teacher training [20], act as industry consultants for learners practising chemical engineering safety investigations [19] and facilitate simulated patient consultations in medical education [21]. In these applications, the chatbot's primary function isn't to retrieve accurate information from course materials but to enable learners to apply their knowledge in authentic scenarios [21]. For example, while an AI tutor might list disease symptoms in medical education, an AI role‐play would simulate a patient consultation where learners diagnose the disease based on presented symptoms [22]. Significantly, this approach mitigates the issue of hallucinations found in AI tutors by allowing for simulated human interactions where uncertainties and occasional mistakes are natural occurrences [23]. Consequently, we argue that errors in AI role‐play are sometimes less critical, and that it is more feasible to simulate an authentic human conversation (through AI role‐play) than to simulate an infallible omniscient (through AI tutors).

In dentistry, simulation is a form of experiential learning that provides students with the opportunity to acquire or improve application of knowledge and skills in a controlled environment. Simulation activities play a vital role in preparing students for clinical practice. Current simulation methods, such as role play, standardised patient actors, and high‐fidelity mannequins, allow students to practice clinical reasoning, communication skills, and decision‐making processes, enhancing their preparedness for clinical practice [24]. However, some of these methods can be expensive, logistically complex to coordinate, and lack the flexibility to be used in traditional classroom settings.

In designing learning experiences for a classroom setting, particularly those involving emerging technologies like AI, it is essential to consider established educational theories. Constructivism serves as a prominent theoretical framework to foster problem solving and critical thinking skills in students. Rooted in Vygotsky's sociocultural theory, constructivism posits that learners actively construct their own understanding of the world through experience [25, 26]. This approach emphasises the importance of social interaction, dialogical learning, and student autonomy in the meaning making process [25]. Central to constructivism is the principle that learning is most effective when learners engage in activities that require them to actively construct meaning from their experiences, and as a pedagogy, it underpins various student‐centered teaching methods including flipped classroom models and active learning strategies [27].

Thus, the emergence of GenAI for AI role‐play presents some promising opportunities for education. First, it can enable students to construct meaning through real‐world application and tasks. Second, it offers a feasible method for incorporating simulation activities into traditional classroom settings [28].

This study aimed to design and implement an AI role‐play activity in a classroom setting and to evaluate students' perceptions of the activity to inform future improvements to the chatbot design. The specific objectives were to:

1. Design an AI role‐play involving a virtual patient experiencing dental pain.
2. Implement the AI role‐play activity in a classroom setting with dental students at different levels of training.
3. Evaluate student perceptions of the usefulness and design of the activity.

## 2 Materials and Methods

We employed a design‐based research (DBR) approach, which allows for iterative cycles of design, implementation, and evaluation of educational interventions in real‐world classroom settings [29]. We opted for DBR due to its emphasis on the systematic design and redesign of educational interventions, particularly ones involving technology. This study represents the first few iterations of the activity, where we describe the initial piloting and testing of the AI role‐play in a classroom setting and design improvements to optimise the activity, establishing a foundation knowledge for subsequent cycles of refinement and evaluation.

The AI roleplay was implemented with second and final year students in an Australian Bachelor of Oral Health program. Students were recruited via convenience sampling. In our program's curriculum, students encounter varying levels of clinical responsibility. In year 1 of the program, they observe clinicians treating patients alongside learning basic sciences and procedural skills. In year 2, students commence working with patients early in the academic year, whereas the final year is predominately workplace‐based learning. During all clinical sessions, students are supervised by clinical educators. Students in different years of the program have different needs, with second year students needing learning experiences that support their transition from simulation to working with patients for the first time, whereas final year students need support consolidating skills or revisiting skills that they have not encountered frequently during clinical practice. Traditionally, in‐class activities to support students learning can include case discussions, treatment planning seminars, and case‐based learning, with students applying their patient history taking skills during peer‐to‐peer role playing exercises.

### 2.1 Case Scenario Development and Chatbot Design

In this study, constructivism provided a theoretical lens for the development of the AI role‐play. It was essential that the scenario mirrored authentic clinical encounters that students might experience. In dentistry, patients presenting with dental pain are a common yet challenging encounter for clinicians, and research has highlighted a need to better support students in assessing and managing patients presenting in pain [30]. When a patient presents in pain at the dental teaching hospital, students are typically expected to first undertake a systematic medical and pain history for the patient and then have a discussion with their clinical supervisor to discuss key findings and diagnostic tests indicated based on the presenting complaint.

The case scenario created was based on an actual patient who presented with two complaints of pain. The first was odontogenic pain: a short, sharp pain to cold stimuli, ceasing on removal of the stimulus, associated with the upper right lateral incisor. The second complaint was neuropathic pain, where the patient perceived feelings of tingling and loss of sensation on the left‐hand side of the face. While the identification of the root cause of the latter problem was outside the scope of practice for the students, it was expected that they would be able to undertake a systemic pain history, identify two causes of pain, and request the appropriate diagnostic tests as part of the immediate management for the simulated patient. The patient had provided informed consent to use their history, imaging and diagnostic test results to develop AI role‐play tools to support students' clinical reasoning.

To simulate the clinical scenario of managing a patient presenting with pain, the role‐play activity was structured into multiple elements, mirroring the typical workflow in the dental hospitals teaching clinics:

1. Patient history: Students began by conducting history‐taking with the AI‐simulated patient, who presented with the previously described complaints of odontogenic and neuropathic pain (Data S1).
2. Information synthesis: Following the history‐taking, students were required to synthesise the gathered information, formulating a preliminary assessment of the patient's condition.
3. Diagnostic planning: Based on their assessment, students then needed to recommend appropriate diagnostic tests, preparing a rationale for each test selected.
4. Supervisor interaction: Students then engaged with an AI‐simulated clinical supervisor, presenting their findings, proposed diagnostic tests, and justifications for their choices. This interaction allowed students to practice their clinical reasoning and communication skills (Data S2).
5. Test retrieval and interpretation: As an additional design element, the AI supervisor could retrieve and display the diagnostic tests requested by the students. These tests included a range of multimedia elements such as:
  - Diagnostic images (panoramic, bitewing, and periapical radiographs).
  - Clinical photographs.
  - Videos demonstrating cold and percussion tests.

This simulation allowed students to experience the initial patient interaction through to diagnostic planning and supervisor consultation. The inclusion of retrievable diagnostic tests added a layer of realism and allowed students to practise interpreting clinical data in context.

To reinforce the constructivist learning design, the AI patient was designed to simulate history taking with an actual patient; patients can be reticent, unclear, or unreliable in discussions [31], and the patient history interview should reflect this. This allowed students to apply their knowledge to make new connections, develop critical thought (what questions should be asked), and develop clinical empathy or ‘bedside manner’ (how the questions are asked). We did this by limiting the amount of information the chatbot provided per reply, and inputting expectations or personal traits from the patient.

For the simulated clinical supervisor interaction, the activity deviated from an authentic clinical interaction. The AI supervisor was designed more like a ‘Socratic tutor’ to draw out the student's learning [32], so the AI supervisor might ask students questions, and probe them further for justifications. This may be distinct from a natural clinical environment where time pressures might mean a supervisor provides a diagnosis (skipping the opportunity for student learning) or would quickly overrule an incorrect diagnosis (in the interests of patient safety). We selected this approach to allow students to actively construct their knowledge by directly applying it (identifying information needed for collection or making a referral), framing it through discourse (describing the course of action and reasons with the AI supervisor), and then reflecting on their understanding (when the AI supervisor provides leading feedback). This could also instigate another iterative round of questioning (the AI supervisor's reply leads to another idea or line of questioning).

The system comprised two interconnected chatbots, built using Streamlit for the front‐end user interface. Streamlit, an open‐source framework that uses Python scripts, required specific backend code to deploy the chatbot and manage API fetch requests, enabling user input to be processed through the large language model (LLM) and generating a corresponding text response. For instance, Python scripts defined the layout of the chatbot, including the landing page, sub‐menus, and the integration of diagnostic images in the supervisor chat. These scripts also managed the data flow between the user interface, where students input their queries, and the LLM, which generates a response.

**FIGURE 1:**
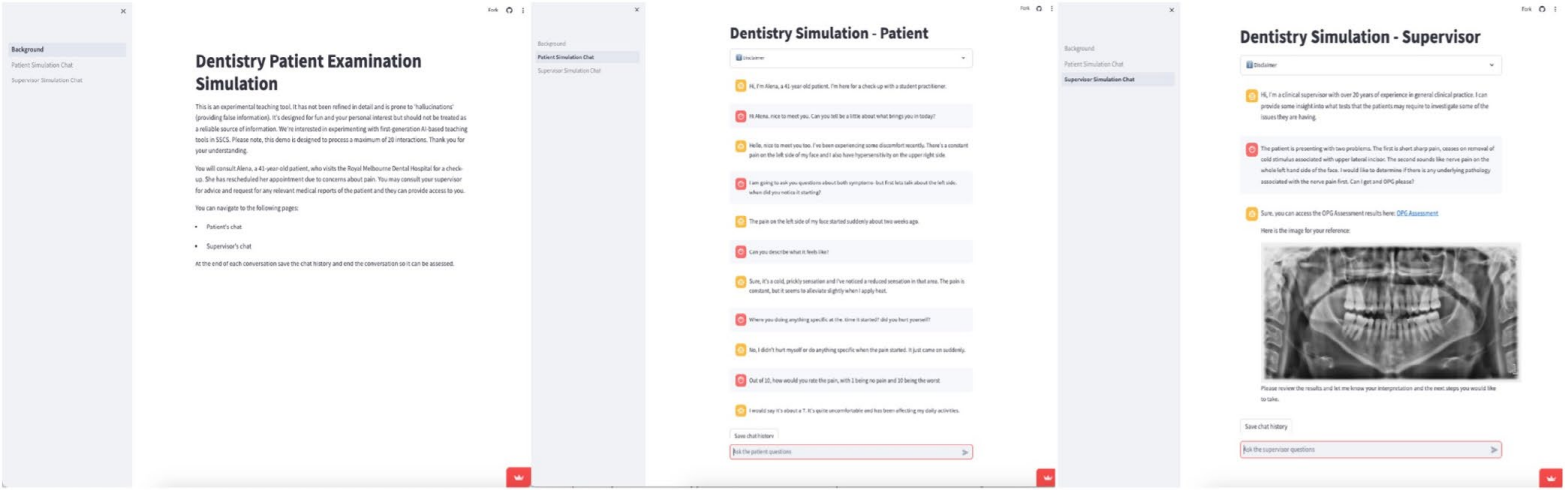
Chatbot Interface. From left to right, this figure depicts the introduction screen, the AI patient interface, and an example of the AI supervisor interface.

Initially powered by GPT‐3.5 Turbo and later upgraded to GPT‐4, the chatbots interacted with students through a unified platform. The patient chatbot, representing a 41‐year‐old individual named Alena, responded to student inquiries about symptoms and medical history (Figure 1). In contrast, the supervisor chatbot, embodying a dental professional with over 20 years of experience, guided students in diagnosis and provided access to diagnostic test results (Figure 1).

Careful prompt engineering was employed to define roles, constraints, and available information for each chatbot. The system utilised different ‘temperature’ settings(a parameter controlling response randomness in AI models)with 0.1 for the patient and supervisor, mimicking the variability in patient responses and the consistency expected amongst clinical supervisors. The system integrated a range of simulated diagnostic tests, including radiographs and clinical examinations, which were embedded directly into the chat interface. Details of the Chatbot system have been uploaded onto the open‐source platform GitHub [33].

### 2.2 Testing and Implementation

Before broader implementation, the AI role‐play underwent alpha testing by the research team, focusing on response accuracy, context maintenance, and user interface functionality. Subsequent beta testing with 12 faculty members and two students assessed clinical authenticity, educational value, and technical performance. Feedback from these phases led to refinements in dialogue patterns, user interface enhancements, and optimisations in diagnostic test result retrieval, ensuring a robust and educationally sound system.

In April 2024, the AI role‐play was implemented with 46 oral health students during a 60‐min classroom tutorial. The activity was scaffolded alongside their pain and pain management theoretical component. These learners were considered novice clinicians and had just commenced seeing patients in the dental teaching clinic. The learning objectives for this session were for students to apply a systematic approach to taking a pain history and to provide a rationale for and to be able to interpret diagnostic tests. Students were given the option to work as groups or as individuals. Students were instructed to document a concise summary of their findings from the history and to interpret and record findings for each diagnostic test as they would in their clinical records. The information was recorded in a Google document, which the educator used to inform discussion points during the tutorial. The educator manually reviewed the Google documents to generate student feedback after the tutorial to be provided to students in a subsequent lesson. The constraints of this approach were that a debriefing session could not be initiated on the same day as the activity. After this activity, the AI role‐play was upgraded to use GPT 4 and a mechanism to save the students interactions with the chatbot, so they could be viewed by the educator to facilitate debriefing.

In July 2024, the AI role‐play was tested with 24 final year oral health students during a seminar. These students were more experienced in seeing patients and were more autonomous in their decision‐making. The learning objectives for this activity were for students to determine the appropriate management pathways for the simulated patient. These students were instructed to work individually and given a time limit of 20 min to engage with the activity to understand if they could work within set time frames. Students were asked to save their chat histories in the browser. After this session, the research team used saved chats from the AI role‐play and ran them through GPT to summarise how the students interacted with the AI role‐play (Data S3). This enabled the educator to understand how the students were interacting with the chatbot and was faster than manually reviewing the saved histories. A limitation of this approach was that it could not be applied in real time.

### 2.3 Data Collection and Analysis

Educators facilitating the classes for both year 2 and final‐year students recorded real‐time observations on student interactions, question patterns and overall engagement. Notes were collated into a summary document after each session to inform early‐stage design improvements.

A previously validated usability survey, originally developed for use in serious gaming contexts, was conducted with final year students to evaluate the usability of the educational tool [34] using the online Qualtrics platform. A 5‐point Likert scale was used to respond to each question, with the options corresponding to the following scores: 1 for ‘Strongly Disagree,’ 2 for ‘Disagree,’ 3 for ‘Neutral,’ 4 for ‘Agree,’ and 5 for ‘Strongly Agree.’ The survey also included a text box where students could enter any additional comments they had about the activity. Only completed questionnaires were considered for analysis. A content analysis of the free text data was undertaken. The descriptive analysis is presented, and figures were generated using RStudio [35].

To evaluate the feasibility of implementing the AI role‐play activity, we also monitored the computational costs associated with using the ChatGPT‐powered chatbots for the final‐year students during a 20‐minute classroom activity.

### 2.4 Ethical Considerations

This study received ethical approval from the relevant institutional board to implement case learning using digital technologies and collect data on student engagement and feedback (Human Research Ethics Committee no. 28062). All participating students provided informed consent before engaging in the study and completing the feedback survey. Explicit written consent was obtained from the patient for use of their history and records for the purpose of education and research.

To use GenAI in the classroom, we sought additional approval from the relevant Associate Dean within our faculty for institutional permission to proceed. To minimise risks to students, we designed the learning activity to avoid collecting any identifying student data. We deployed an open‐access user interface that did not require any sign‐in, and students were instructed not to enter any personal information. These strategies were consistent with a previous use case where a Privacy Impact Assessment deemed similar activities below the threshold for additional institutional review [19].

## 3 Results

### 3.1 Educator Observations

The ability of the activity to adapt well to different instruction and learning objectives across year levels highlights potential multiple use cases for a single case scenario.

During the first iteration of the AI role‐play, the second‐year students were asked to participate in the activity and advised they could work as small groups or individuals. The educator reported all students opted to work together, except for a single student who chose to work alone. They documented ‘Most students appeared engaged, actively discussed the case, and deliberated on what questions to ask the AI patient’. However, they documented initially ‘some students approached the AI role‐play with overly simple or direct questions, rather than using a natural conversational style’. To address this, the educator prompted the students to treat the activity as a real patient conversation. Additionally, although most students opted to work as groups, the educator observed several students who did not engage in much discussion and instead opted to communicate only through the shared Google document. The educator also reported ‘There were some disagreements on how to approach history taking. Some were resolved through discussion, with students referring to their learning materials, while others were leveraged for class discussion’. The educator reported that some student groups focused on specific and narrow lines of questioning with the AI patient, while others applied a more systemic and structured approach to their questioning. The educator perceived that the activity helped them identify gaps in the student knowledge. Those that were noted were that students needed to be more systematic in history taking, they needed support for justification of appropriate diagnostic tests contextualised to the needs of the AI patient, and they needed guidance for interpreting tests and record keeping. The educator reported that during this activity students would have benefited from templates or examples of exemplar reports to help guide their record keeping.

In the second iteration of the AI role‐play, the educator noted that students appeared excited and enthusiastic about the novelty of the activity. The educator reported the final year students were able to ‘work swiftly, with a structured approach to their line of questioning… and appeared confident in selecting appropriate diagnostic tests for the patient’. The educator noted final year students still required support for interpretation of the tests and for clinical decision‐making. They noted that on completion of the activity, ‘…some students organically, without direct instruction, engaged in peer discussion to compare their outcomes’. However, they noted more time should have been allocated within the seminar for discussion and de‐briefing. Whilst it was helpful to view saved versions of the students' interactions with the chatbot, the educator provided feedback to the team that ‘real‐time feedback summarizing students' input into the AI role‐play would have helped them provide ‘timely, tailored feedback’ during class. Without this feedback, students could not gauge if they had appropriately managed the patients' presenting complaints based on the AI supervisor's feedback alone.

### 3.2 Survey Results

The usability survey was undertaken with the final year students. Of the 24 final year students who participated in the AI role‐play, three did not consent to participate in the survey, seven partially completed it, and 14 completed and submitted it. The ages of the students who completed the survey ranged from 18 to 25 (71%) and 26 to 30 (29%).

**FIGURE 2:**
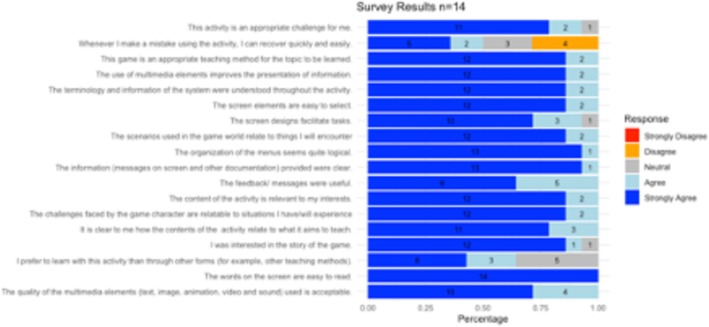
Usability survey results. This stacked bar chart visualises survey results regarding the usability of the AI roleplay. The response key is located on the far right. Each segment of the bars includes the count of responses, except where the count is zero, in which case no number is displayed.

Student survey responses are outlined in Figure 2. The students reported an overall positive response to the screen design elements and user interface for the AI role‐play. All students (100%) strongly agreed the words on the screen were easy to read. Students also strongly agreed that the design of the screen facilitated tasks (71.4%), the screen elements were easy to select (85.7%), the organisation of the menus was logical (92.8%), the terminology used by the AI role‐play was understood throughout the activity (85.7%), and that the feedback messages were useful (64.2%). Most students strongly agreed that multimedia elements improved the presentation of the case information (85.7%) and that the multimedia elements were of an acceptable quality (71.4%). Regarding the message quality, most students strongly agreed that the information provided during the AI role‐play was clear (92.8%). When students made mistakes or errors in their communication, many disagreed (28.6%) or reported a neutral response (21.4%) when asked if they believed they could recover quickly from that error.

Regarding the authenticity of the AI role‐play design, most students strongly agreed that the content was relevant (85.7%), that it related to the teaching aims (78.5%), and that the AI role‐play relates to scenarios (85.7%) and challenges (85.7%) they will encounter in real life.

Students' perceptions of the learning activity showed most students strongly agreed the AI role‐play was an appropriate teaching method (85.7%) and that they were interested in the story of the AI role‐play. However, when asked if students preferred to learn with this activity compared to other teaching methods, there was more variability in student responses, and a third of students disagreed (14.2%) or were neutral (35.7%) in response to this statement.

The students also had the opportunity to provide free text feedback at the end of the survey. The comments were grouped into four categories: the utility of the AI role‐play simulations, learner engagement and enjoyment in the activity, innovation, and suggestions for improvement (Table 1).

**TABLE 1:** Student feedback on AI role‐play is categorised into five groups.

Category	Feedback
Utility of AI role‐play	These simulations were useful and provided me with insight on how I can word my questions to gather more specific information when collecting patient history This is a very effective way to simulate scenarios This is a very effective learning tool because I would like to use ChatGPT to practice my clinical skills and patient case presentation skills but ChatGPT is not a dentistry or medical based model, so this simulation is very useful If this was set as homework more people would participate because it is engaging and interactive I would've found this helpful especially in earlier years when I was beginning to see patients and I would be a little bit awkward when interacting and engaging with patients I believe this chatbot will be very helpful for student self‐learning
Engagement	This is very relevant to my clinical experience. I really like it This was an extremely engaging and beneficial activity to my learning, I had quite a bit of fun and would enjoy more sessions/more cases to practice pain history of patients The activity was very interesting and interactive! This activity is super fun and I think it would be great to make use of this activity for students to practice medical history, pain history taking. I also think it's great that you can discuss with supervisors what diagnostics you need and why they are necessary to help with reaching the right diagnosis. This learning activity is very engaging and a great learning tool
Innovation	I believe these would be practical to my learning and career. Really great advancement of technology/use of AI This is such an innovative tool, and I believe it can help the students fresh into the clinic This is a creative way to get student practitioners to practice engaging with patients. It is quite realistic as well and so it's relevant to clinical practice
Suggested improvements	The speed of reply and comments could be slowed down and summarised. Sometimes the chatbot gave me the answers before they asked a question, therefore I already had the answers. For improvement, would be nice for the simulated patient to be able to ask questions to assist in my understanding of answering. The only advice I would provide is that the supervisor did not provide as much information in the response and if it was more concise. One area this model can improve is that the patient is very keen to provide many details of their history. However, in real life, clinicians often need to dig out all the details. This was a very engaging exercise despite some areas of errors when requesting results during different attempts. Sometimes I would give my rationale for the investigations, but it would ask me again.

### 3.3 Cost Considerations

Over a day in a 20‐min activity with just over 20 students, the token costs were $USD 13.17 (AUD $19.45), corresponding to 408 API requests (about 20 queries per student during the activity). The tokens usage was heavily weighted to text uploads (context tokens, 393 k, information sent to OpenAI) rather than text returned from the chatbot (generated tokens, 23.5 k, information sent back from OpenAI). ChatGPT is stateless, so we needed to reupload the system prompt with each query, which quickly created a large volume of context tokens (uploaded textual information). For our initial study, the usage costs are still low. However, we noted that these could be further reduced by optimising the system prompt to reduce its size while maintaining its functionality and reliability. This would make the tool more cost‐scalable if it were to be implemented across an entire faculty or department.

## 4 Discussion

This study describes the design and initial implementation of an AI role play to simulate a patient and supervisor interaction for dental students. Exploring new use cases for GenAI in dental education requires an agile research approach, involving iterative cycles of implementation, feedback, modification and testing. As technology evolves rapidly, educators must also be nimble, sharing insights and improvements early to prevent designs from becoming outdated. For this reason, this study presents a descriptive analysis of the design and initial implementation and highlights opportunities for design improvements for AI role play in dentistry.

While the AI role‐play activity was informed by constructivist principles, it is the surrounding educational design, including facilitation, student interaction and feedback, and reflection, that situates it within a constructivist learning paradigm. The chatbot acted as a prompt for exploration and discussion, not as a standalone teaching tool. Students described the activity as authentic, engaging and highly relevant. The case design was appropriately challenging, and the implementation created opportunities for peer learning. Prior research has reported that AI chatbots can enhance engagement through interactive and conversational learning [36, 37]. Our preliminary findings reinforce this, with students describing the activity as fun and effective. They also expressed it would support their preparation for clinical practice, consistent with current views that GenAI can assist in transitioning from pre‐clinical to clinical settings and assist with clinical decision‐making in early dental training [38].

Student responses highlighted variability in how students experienced the AI role‐play compared to conventional activities. This contrasts with a recent study of healthcare students who preferred the AI chatbot over traditional role‐plays for practising taking medical histories [39]. This underscores the importance of designing multi‐modal strategies that cater to different learning preferences. AI role‐play may be most effective when embedded within broader learning programmes that blend digital tools and traditional approaches.

Traditional role‐play with actors or peers is well established in medical education and can provide realistic communication challenges, emotional dynamics and opportunities for performance feedback. However, it can be resource‐intensive, difficult to scale, and may vary in standardisation and availability [40]. AI role‐play offers a promising solution for creating more accessible and scalable simulation training. This is especially relevant as dental schools face increasing pressure to deliver diverse and relevant clinical placement experiences to meet both institutional and accreditation requirements. Simulation plays a vital role in preparing students for clinical practice, enabling them to practice reasoning, communication, and decision‐making skills. However, traditional simulation methods can be logistically challenging to scale or use in classroom settings. AI role‐play is relatively low‐cost, flexible, and is not constrained by scheduling and staff‐student ratios, making it a potential option for wider implementation.

Although GPT‐powered chatbots are known for occasional irrelevant responses or inaccurate responses (hallucinations) [41], in our use case these moments led to valuable learning opportunities. For instance, students often pursued narrow or overly detailed lines of questioning, or asked questions not directly related to the specific patient case during history taking. The latter has also been observed in serious games for medical education involving chatbots [42]. In our use case, it resulted in the AI patient providing extraneous information, and as a result some students failed to identify key symptoms such as neuropathic pain. When this was revealed in class discussion, students appeared surprised to miss this detail, and to realise this could delay timely medical care in real clinical settings. This type of trial‐and‐error or struggle‐led learning, where students make mistakes and reflect on them in a safe environment, aligns with the concept of productive failure and supports constructivist pedagogy [43].

Educator observations highlighted the potential of AI role‐play to be used in classrooms for data‐driven feedback. Initially, second year tutorials required the educator to manually collate student interactions with the Chatbot, which was time‐consuming and delayed feedback. For final year students, we introduced a system where chat transcripts could be saved to a database, extracted and summarised using GP to generate feedback based on the student's interactions with the AI patient and AI supervisor. We have since automated this process so the educator can access these summaries on the user interface. We do this by recording the student queries and GPT replies in MongoDB, then feeding the complete transcript into a GPT to summarise the replies. The educator becomes the ‘human in the loop’ who can interpret the summaries and relay feedback to the students, understanding the context of the problem [44]. This example demonstrates how educators, regardless of the class size, could use an AI role‐play and leverage the GPT to augment their instruction and provide personalised, real‐time feedback to students. Additionally, we are also exploring implementing a real‐time analysis tool to prompt students to use more natural, open ended, conversational communication style during their interactions with the chatbot.

Despite the strengths of the AI role‐play, challenges remain. Real patient cases often involve complex medical histories, comorbidities, and unique social and cultural factors influencing clinical presentation and history‐taking. Chatbots can oversimplify these complexities or provide excessive information. This is a common issue with GPTs because they are trained to demonstrate a comprehensive understanding, anticipate user needs, and ensure user satisfaction by being thoroughly informative. In essence, they can be ‘too helpful’ and may not mirror authentic patient interactions [19]. Student feedback also suggested this, noting that the AI patient sometimes volunteered too much information while the AI supervisor felt less responsive. The literature cautions that educational chatbots may cause frustration and irritation to students if they fail to provide meaningful responses [45]. To mitigate this, the AI patient was explicitly programmed to deliver information gradually and only in response to targeted questioning. The prompt instructed the AI to “refrain from giving too much information about your condition, let the student ask questions to uncover your symptoms and condition,” and to “provide one fact at a time and respond to the student's questions.” Despite these efforts, optimising system prompts remains an ongoing challenge.

This study has several limitations. First, it relied on educator observations, which provide valuable domain insights but lack the methodological rigour of focus groups or structured interviews. Future iterations will incorporate more robust qualitative data collection and consider measuring constructivist learning outcomes. Second, as this study is situated within an early DBR cycle, the data analysis was descriptive only, reflecting our focus on gathering insights for iterative design improvements, and the small sample size limits the generalisability of the findings. Third, the tutorial discussions could benefit from more structured debriefing using recognised frameworks. Fourth, we did not explore newer models, such as ChatGPT‐4.0 or 01‐Mini, which could enhance the quality of chatbot interactions. Lastly, a key limitation of using text‐based interactions is the difficulty in conveying nonverbal cues and emotional nuances that are essential in sensitive patient encounters.

Future research directions should aim to increase the versatility, authenticity and efficiency of AI‐generated patient simulations. Creating a library of diverse personas could reduce scenario development time while enhancing case variety. Additionally, varying chatbot communication styles, such as verbosity, pacing and tone, could help support students adapt their own communication approaches to better suit the individual patient. Experimenting with voice‐to‐text functions or emotion‐tagged text responses could create a more immersive and empathetic patient interaction. Furthermore, the versatility of large language models also presents opportunities to simulate diagnostic test outputs alongside patient symptoms. Future research could explore the feasibility of autonomously generating relevant test results. These advancements would significantly expand the educational potential of AI role‐play activities.

While AI role‐play can simulate conversational aspects of clinical encounters, it cannot replicate the physical aspects of patient care, such as performing examinations, procedural skills and behaviour management. As such, GenAI tools should be recommended as a broader suite of educational strategies. Their integration with augmented reality, virtual reality, and adaptive avatars may offer future students opportunities to develop skills in lifelike, adaptive environments, providing real‐time feedback and personalised learning experiences. This raises important questions about when and how these activities should complement or replace real patient interactions. Ensuring that educational quality and patient care standards remain high will be critical, and ongoing research will be essential to guide these decisions in dental education.

## 5 Conclusion

AI roleplay using GPT‐powered chatbots has a potentially unique role in preparing students for clinical practice. This study applied a constructivist framework to guide the design of the AI role‐play for oral health students. The outcome was an engaging and authentic learning activity. Educator and student feedback have informed design improvements and provided valuable insights into how AI role‐play can be developed to augment teaching practices and improve student learning outcomes in dental education.

## Conflicts of Interest

The authors declare no conflicts of interest.

## Supporting information


Appendix S1.

## Data Availability

The data supporting this study's findings are available from the corresponding author upon reasonable request.
