# Supplementary material for: Artificial Intelligence Chatbots as Virtual Patients in Dental Education: A Constructivist Approach to Classroom Implementation
Source: Eur J Dent Educ. 2025 Jun 3;30(2):447–56. doi: 10.1111/eje.13135 (PMC13090419; doi:10.1111/eje.13135)
Supplement: Supplementary file 1 — Appendix S1. [file EJE-30-447-s001.docx]

**Supplementary Materials**

**Supplementary Material 1: Screenshots of the AI Role-Play patient interface**

**
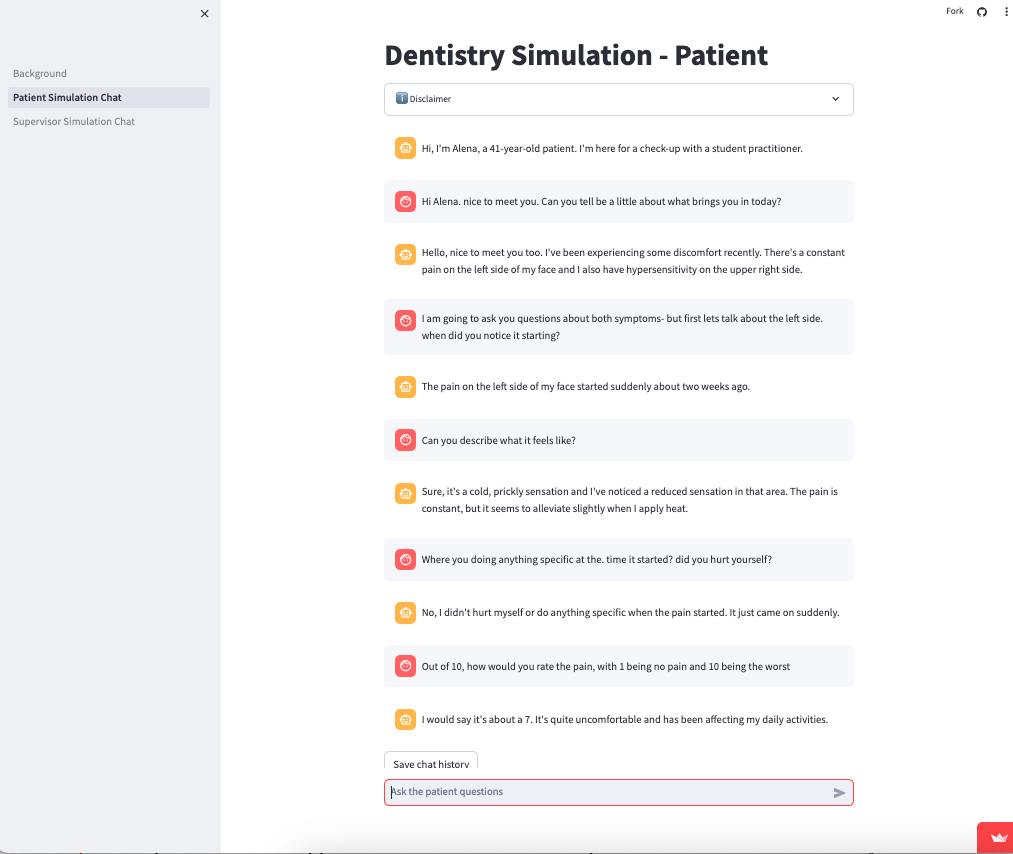
**

**Supplementary Material 2: Examples of the Questions that the AI-Supervisor may ask to probe students.**

**AI role-play supervisor’s probing questions and prompts**

These questions were designed to guide student reasoning, reinforce clinical thinking, and encourage justification of tests.

**History & Symptoms**

“Can you please provide a summary of the key points from the patient's history?”

Asked repeatedly to prompt clinical reasoning based on information gathered.

“What signs or symptoms led you to this provisional diagnosis?”

To justify a diagnosis like reversible pulpitis or non-dental origin.

**Test Justification & Interpretation**

“Which type of radiograph are you considering and why?”

“Can you please explain why you've chosen these tests and how you expect them to contribute to your diagnosis?”

“Why did you choose this specific test?”

These were used after students requested bitewings, periapicals, OPGs, or vitality tests.

“What are you hoping to find or rule out with these tests?”

Encouraging students to define the clinical question behind each test.

“Do you think any additional tests are required based on these results?”

To assess clinical completeness after reviewing radiographs or cold/TTP tests.

**Critical Thinking / Provisional Diagnosis**

“Could you explain your rationale for choosing this test?”

Especially used with CO₂ test, cold testing, or referral for OPG.

“What would be your provisional diagnosis based on the findings?”

**Encouraging students to commit to a diagnosis and use supporting evidence.**

“What other causes might explain the symptoms?”

When radiographs showed no caries or pathology.

**Patient Management Planning**

“What would be your proposed management plan for this patient?”

“Are there any other diagnostic or referral steps you would take?”

**Supplementary Material 3: GPT summary of student performance**

**Strengths Observed**

1. Rapport Building & Consent

- Most students introduced themselves and confirmed consent for history taking, examinations, and radiographs.
- Some acknowledged supervision and explained procedures, supporting patient trust.

1. Medical History & Medication Inquiry

- Nearly all students consistently asked about:
  - Medical conditions (anxiety, reflux).
  - Medications (Lexapro, Nexium).
  - Allergies (penicillin) and reactions.
- A few asked for dosages and duration of medications, but not all.

1. Chief Complaint Exploration

- All students elicited the main complaints left facial pain and right-sided cold sensitivity.
- Most asked about onset, location, triggers, and character of the pain (e.g., sharp vs dull).

1. Pain Details

- Students probed into:
- Duration (2 weeks for left-sided pain).
- Nature of pain (cold, prickly).
- Relief measures (heat).
- Impact on sleep and daily life.

1. Oral Hygiene & Habits

- Some students asked about brushing, flossing, and diet.
- A few explored clenching/grinding

**Commonly Missed or Incomplete Areas**

1. Inconsistent Exploration of Cold Sensitivity

- Few students clearly differentiated the cold pain on the upper right from the constant pain on the left.
- Often grouped together or missed the chronicity of the cold sensitivity (present for over a year).

1. Lack of Specificity in Pain Location

- Several students did not ask the patient to point to a specific tooth or area.
- This led to vague localization ("left side of the face") persisting across assessments.

1. Failure to Clarify Triggering & Lingering of Symptoms

- For cold sensitivity, only some clarified:
- Is the pain lingering?
- How long does it last after the stimulus?
- Is it spontaneous or always stimulus-driven?
- Important for distinguishing reversible vs irreversible pulpitis.

1. Limited Follow-up on Medical History

- Students did not explore:
- Impact of anxiety or reflux on oral health (e.g., bruxism, erosion).
- Any other medications or supplements.
- Management of penicillin allergy beyond “I avoid it.”

1. Incomplete Pain Impact Assessment:

Few asked about:

- Impact on eating or daily life.
- Quality-of-life implications.
- Emotional stress from symptoms.

1. Limited Review of Dental History

- Most noted braces or frenectomy but failed to ask:
- Any past trauma, restorations, or root canals?
- Were there revious symptoms or episodes in this region?

**Recommendations for Improvement**

| Area | Actionable Suggestion |
| --- | --- |
| Symptom Clarity | Use structured questioning: “Where is the pain? When does it start? What makes it better/worse? Does it linger?” |
| Pain Mapping | Ask patient to point or describe *specific teeth* or regions affected. |
| Chronology | Establish timelines separately for each complaint (e.g., acute left pain vs chronic right-side sensitivity). |
| Oral Habits | Ask routinely about clenching, grinding, or habits linked to anxiety. |
| Medical Relevance | Explore how systemic conditions or medications may affect dental symptoms (e.g., reflux causing erosion). |
| Red Flag Awareness | Consider neuropathic or non-dental causes for diffuse, cold-type, non-stimulus triggered pain. |
| Documentation Routines | Ensure students check when last x-rays were taken and if any prior diagnostic steps were done. |
